# Dos and don’ts in designing a computerized oral and lip squamous cell cancer registry

**DOI:** 10.1186/s12913-023-09860-3

**Published:** 2023-09-19

**Authors:** Leila Shahmoradi, Nazanin Mahdavi, Hana Saffar, Reza Ghalehtaki, Mohammad Shirkhoda, Maziar Motiee-Langroudi, Mohammad Javad Kharazi Fard, Sorayya Rezayi, Erfan Esmaeeli

**Affiliations:** 1 Health Information Management and Medical Informatics Department, School of Allied Medical Sciences, Tehran University of Medical Sciences, Tehran, Iran; 2 Department of oral and maxillofacial pathology, School of dentistry, Tehran University of Medical Sciences, Tehran, Iran; 3 Cancer institute, Imam Khomeini hospital complex, Tehran University of Medical Sciences, Tehran, Iran; 4 Radiation Oncology Research Center, Cancer Research Institute, Tehran University of Medical Sciences, Tehran, Iran; 5 Department of Radiation Oncology, Cancer Institute, Tehran University of Medical Sciences, Tehran, Iran; 6 Cancer Research Center, Cancer Institute of Iran, Tehran University of Medical Sciences, Tehran, Iran; 7 Imam Khomeini hospital complex, Tehran university of medical sciences, Tehran, Iran; 8 Dental Research Center, School of dentistry, Tehran University of Medical Sciences, Tehran, Iran

**Keywords:** Disease registry systems, Minimum Data Set, Carcinoma, Delphi technique

## Abstract

**Background:**

In the last ten years, many countries have started to develop constructive systems for registering common diseases and cancers. In this research, we intended to determine and identify the minimum data set (MDS) required for the design of the oral and lip squamous cell cancer registration system in Iran.

**Methods and material:**

At first, primary information elements related to disease registries were extracted using scientific papers published in reliable databases. After reviewing the books, related main guidelines, and 42 valid articles, the initial draft of a researcher-made questionnaire was compiled. To validate the questionnaire, two focus group meetings were held with 29 expert panel members. The final version of this questionnaire was prepared by extracting different questions and categories and receiving numerous pieces of feedback from specialists. Lastly, a final survey was conducted by the experts who were present at the previous stage.

**Results:**

Out of 29 experts participating in the study, 17 (58.62%) were men and 12 (40.37%) were women. The age range of experts varies from 34 to 58 years. One hundred-fourteen items, which are divided into ten main parts, were considered the main information elements of the registry design. The main minimum data sets have pertained to the demographic and clinical information of the patient, information related to the consumed drugs, initial diagnostic evaluations of the patient, biopsy, tumor staging at the time of diagnosis, clinical characteristics of the tumor, surgery, histopathological characteristics of the tumor, pathologic stage classification, radiotherapy details, follow-up information, and disease registry capabilities. The distinctive characteristics of the oral and lip squamous cell cancer registry systems, such as the title of the disease registration programme, the population being studied, the geographic extent of the registration, its primary goals, the definition of the condition, the technique of diagnosis, and the kind of registration, are all included in a model.

**Conclusion:**

The benefits of designing and implementing disease registries can include timely access to medical records, registration of information related to patient care and follow-up of patients, the existence of standard forms and the existence of standard information elements, and the existence of an integrated information system at the country level.

## Introduction

Oral cancer is one of the most common malignant cancers among head and neck cancers, which are the sixth most common cancers in the world [1]. Oral cavity and lip cancer are not among the top 10 cancers in Iran [2]. Although a study by Khanali and Kolahi [3] revealed that the incidence rate of oral cancer decreased from 2000 to 2016, some studies reported its increasing trend, especially in individuals over 65 years old [4–7]. Oral cancer accounts for about 37% of head and neck cancers, which account for more than 500,000 cases globally and are expected to increase by 62% to 856,000 cases by 2035 [8]. However, approximately half of all oral cancer patients are discovered at an early stage and achieve great outcomes [9]. India, the “oral cancer capital of the world”, has the highest incidence of oral cancer of any country in the world [10]. It is estimated that there are around 450 new cases of oral cancer diagnosed each year in Iran, with age-standardized mortality rates (ASMR) of 0.7 for males and 0.6 for females per 100,000 people [11]. Buccal carcinoma, gingival carcinoma, maxillary sinus carcinoma, tongue cancer, and carcinoma of the floor of the mouth are the numerous types of oral cancer, 90% of which are squamous cell carcinoma [12]. According to 2020 global cancer statistics, 177,757 people died from cancer in these parts of the oral cavity. Among all cancers, lip and oral cancer rank 20th and 22nd for morbidity and mortality in the Iranian population, with 10,139 new cases and 454 deaths [13]. Generally, excessive smoking, drinking, betel nut chewing, gene mutation, human papillomavirus infection, epigenetic modification, and other internal and external factors are risk factors for oral cancer [14]. Despite the fact that low-income countries have only 57% of all cancer diagnoses, they account for 65% of all cancer-related mortalities. 65% of all cancer-related fatalities occur in low-income nations, while only 57% of cancer incidence occurs globally. Cancer cases in Iran have seen a noticeable increase in the past few years. This trend is expected to continue until 2025, which will significantly burden the country’s healthcare system. To address this issue, it is crucial to implement effective cancer control programs and registries to detect cancers, particularly oral cancer, at an early stage. This will require a tailored approach to meet the population’s specific needs [15, 16].

In order to offer researchers and policymakers confidential information on the incidence of cancer and help them better prepare for and manage its effects, cancer registration is essential in view of the growing cancer burden in developing countries [17].

In high-income countries, technological progress and the value of registries have led to an increase in the quality and use of data over the past few decades. However, in many developing countries, high-quality cancer data is not available due to a lack of health resources and competing priorities. Population-based cancer registries (PBCRs) are a vital component of any national cancer control program that aims to offer vital data on cancer incidence, survival, and death, as well as serve as a helpful conduit for cancer research and a tool to promote cancer management [18]. Population-based cancer registration can be used to monitor the outcomes of initiatives for cancer prevention, early detection or screening, treatment, and palliative care, as well as to assess the size of the cancer burden and its likely future evolution. It also serves as a foundation for research on cancer causes and prevention [19].

There are some works on oral cavity cancer and associated registry systems that have contributed in certain ways. A study by Ben Nasir et al. revealed that Libya developed an oral cancer registry system in 2014 with the following objectives: (1) To compare oral cancer cases mentioned in published publications with other cases of the disease in the area. (2) to outline the early stages of development and long-term objectives of a population-based oral cancer tumor registry system in Libya, which may be used to collaborate with other national, regional, and global population-based cancer tumor registry systems in the future. (3) recommendations that will be required soon for population-based registries in Libya and as a current registry system to describe oral cancer disease patterns and risk factors and if prevention and treatment are needed in that country. The findings of this study indicate that the Libyan national cancer registry program, which envisions five cooperating regional cancer registries, is still working at a poor level [20]. A study was conducted in the United States in 2013 to describe the demographic and clinicopathological characteristics of oral squamous cell carcinoma (OSCC) diagnosed in oral pathology services in southeastern Brazil over a period of 8 years [12]. In 2010, oral squamous cell carcinoma (SCC) research was conducted in Australia with the aim of investigating the five-year survival and recurrence of oral SCC following incisional vaccination biopsy using data from the Western Australian Cancer Registry. This study demonstrated that oral SCC biopsy may be a safe technique by proving that the kind of biopsy was not associated with the survival of oral SCC patients with stage I or stage II disease [21].

The Iranian Ministry of Health has been operating a national cancer registry program based on pathology since 2000, encompassing each of the country’s 31 provinces [3]. There was no registry system specifically for oral and lip squamous cell carcinoma. Due to the absence of an oral and lip squamous cell carcinoma registration system in Iran, it is very difficult to get information. Moreover, all the demographic, clinical, and paraclinical information, treatment and follow-up information, and pathology reports are documented in a disorganized and inconsistent manner, making it extremely difficult and sometimes impossible to access all of this data simultaneously. The systematic collection of data in a large database provides a suitable platform for conducting related research [22].

Therefore, the design, evaluation, and implementation of an oral and lip squamous cell cancer registration system will be one of the best solutions in the field for properly managing this type of cancer. The aim of the current research is to identify and extract the minimum data set for designing an oral and lip squamous cell cancer registration system.

## Method and materials

This is an applied-developmental study carried out at Tehran University of Medical Sciences. In this research, it is intended to determine and identify the minimum data set for designing a registration system for oral and lip squamous cell cancer.

### Extracting primary information elements based on literature surfing

**Fig. 1:**
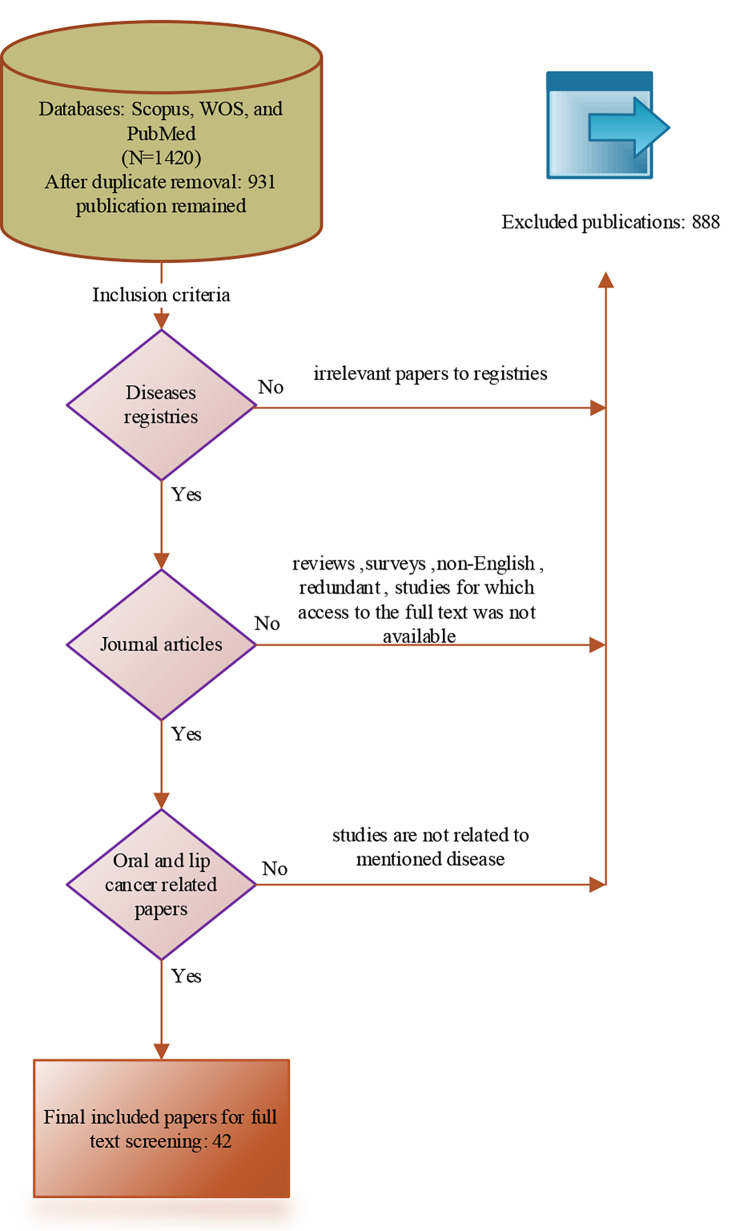
Screening phase of papers

Primary information elements related to disease registries were extracted using scientific papers published in reliable databases such as Medline (through PubMed), Web of Science, and Scopus, a review of websites of similar disease registries around the world, an evaluation of existing patient records, and also the opinion of the research team. Scientific databases were searched from inception to 2022 by combining the words “oral and lip cancer”, “registry system”, and " disease registries”. The Preferred Reporting Items for Systematic Review and Meta-analysis (PRISMA) statement provides the foundation for papers’ screening [23]. Three of the books, including the International Classification of Diseases, 3rd edition (ICD-O-3), Facility Oncology Registry Data Standards (FORDS), Revision of 2016, and International Agency for Research on Cancer (IARC), were used to extract information that was examined by specialists. The flowchart of the screening phase of papers is depicted in Fig. 1.

### Designing a researcher-made questionnaire for conducting a survey

Thereby, after reviewing the beforementioned books, related main guidelines, and 42 valid articles, after several meetings, an initial draft of a researcher-made questionnaire was compiled to determine the minimum data for oral cancer registration. The initial draft of this questionnaire consists of twelve parts including (1) demographic and clinical information of the patient; (2) information related to the consumed drugs; (3) initial diagnostic evaluations of the patient; (4) biopsy; (5) tumor staging at the time of diagnosis; (6) clinical characteristics of the tumor; (7) surgery; (8) histopathological characteristics of the tumor; (9) pathologic stage classification; (10) radiotherapy details (if done); 11) follow-up information; and 12) disease registry capabilities.

#### Validating the designed questionnaire

To validate the questionnaire and finalize it, two focus group meetings were held with 29 expert panel members. The final version of this questionnaire was prepared by extracting different questions and categories and receiving numerous pieces of feedback from specialists in oral and maxillofacial pathology, oncology, general pathology, radio-oncology, maxillofacial surgery, statistics, and epidemiologists. Also, its validity was checked and confirmed by a group of experts, and Cronbach’s alpha was used to check its reliability, which was calculated at 0.85. To determine the content validity of the questionnaire, two categories of questions were answered by 29 specialists; in the content validity ratio (CVR) questions, based on a three-point scale, it was determined whether the provided information elements were “necessary” or “useful but not necessary” or “not necessary”. The content validity index or CVI was also used to measure the validity of the questionnaire. To calculate the CVI, experts were asked to rate the relevance of each item on the following four-point scale: not relevant, in need of major revision, relevant but in need of revision, and fully relevant. The number of experts who chose options 3 and 4 is divided by the total number of experts. If the resulting value is smaller than 0.7, the item is rejected, if it is between 0.7 and 0.79, it should be revised, and if it is larger than 0.79, it is acceptable. After completing the above steps and summarizing the opinions of the research team, the questionnaire items were corrected, clarified, and finalized.

### Identifying and selecting the minimum data set and capabilities

**Fig. 2:**
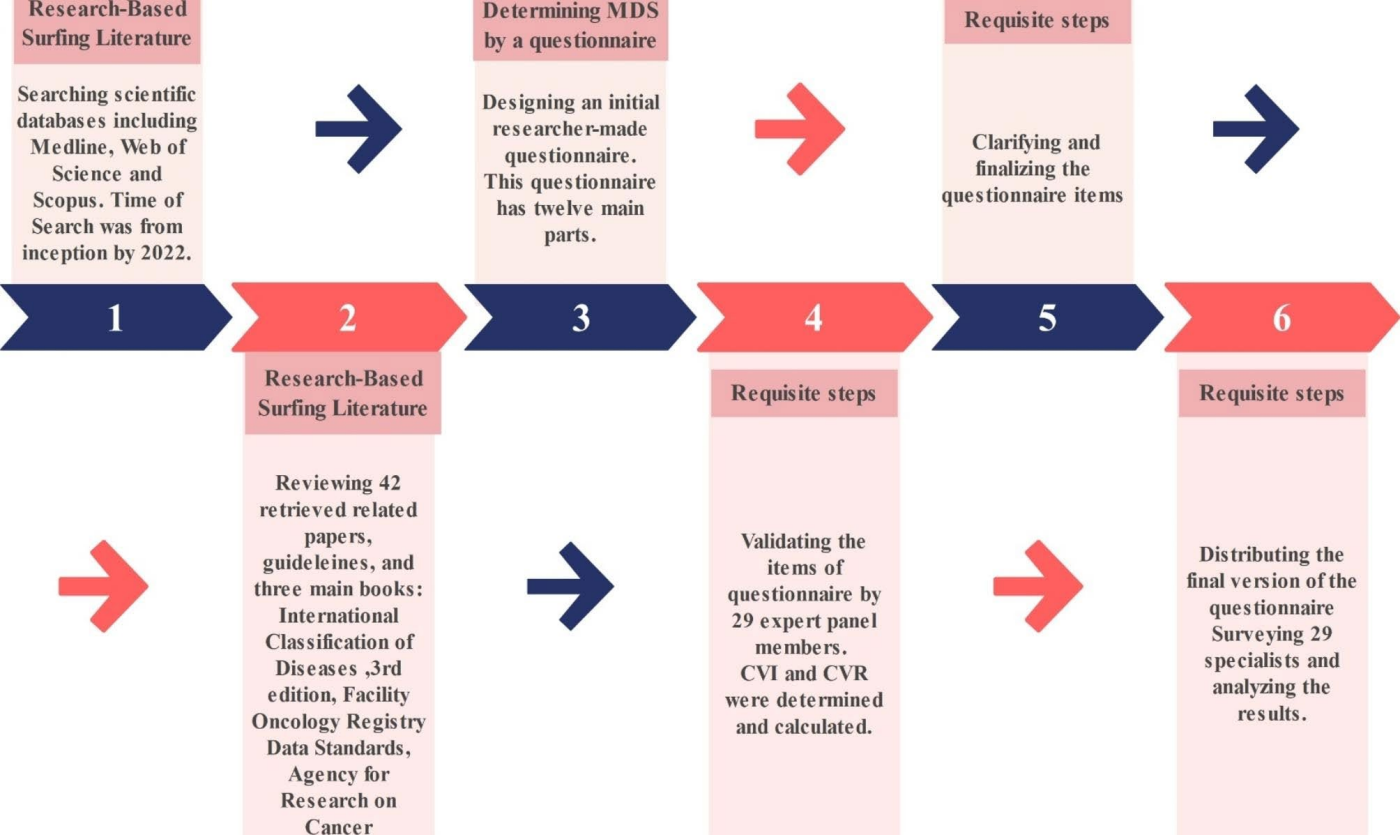
Main steps of the conducted methodology

In the last stage, a final survey was conducted by the experts who were present in the previous stage. The final questionnaire was distributed with the title “Questionnaire for determining the minimum data required for the design of the oral cancer registry.“ The sampling method used in this research was available sampling. The final questionnaire was sent in person and via email. After analyzing the results obtained from the questionnaires collected by the statistical analysis software SPSS version 26, the structural content of the software was extracted. Using the Delphi method, each of the information elements in the questionnaire was evaluated on a scoring scale of one to four based on the Likert scale, and they were considered essential items only if more than 70% of the corresponding points (2 5 out of 4). Information elements that scored less than 2.5 (less than 2.5 out of 4) were considered unacceptable elements and were removed from the set of information elements. Also, in each section after the information elements, an empty section was considered so that the doctor completing the questionnaire could add another element in addition to the mentioned items, according to his discretion. The main steps of the method are depicted in Fig. 2.

## Results

### Demographic information of experts participating in the survey to identify information needs

Out of 29 experts participating in the study, 17 (58.62%) were men and 12 (40.37%) were women. The age range of experts varies from 34 to 58 years. Table 1 shows the descriptive information of specialists by gender, expertise and work experience. As can be seen, eight of the participants were specialists in oral and maxillofacial radiology, five of them were men and three were women. Furthermore, the working experience of 18 specialists is 5 to 20 years, nine of them have 5 to 10 years and also nine have 10 to 20 years of experience. The working experience of only three of the specialists below is 5 years.

**Table 1:** Descriptive information of the participants in the survey

Row Labels: sex, work experience, and specialty	Frequency	Frequency percentage (%)
**Female**	**12**	**41.3**
**More than 20 yrs**.	**3**	**10.3**
Pathologist	1	3.4
Oral and maxillofacial pathologist	1	3.4
Oral and Maxillofacial radiologist	1	3.4
** 5–10 yrs.**	**4**	**13.7**
Oral and Maxillofacial pathologist	3	10.3
Oral and Maxillofacial radiologist	1	3.4
** 10–20 yrs.**	**3**	**10.3**
Specialist in oral diseases	2	6.9
Oral and Maxillofacial radiologist	1	3.4
** Less than 5 yrs.**	**2**	**6.9**
Oral and Maxillofacial radiologist	2	6.9
**Male**	**17**	**58.6**
** More than 20 yrs.**	**5**	**17.2**
Oral and maxillofacial surgery	1	3.4
Oral and maxillofacial surgery, cancer surgery fellowship	1	3.4
Radiotherapy specialist	1	3.4
Radiology specialist	1	3.4
Otorhinolaryngologist	1	3.4
** 5–10 yrs.**	**5**	**17.2**
Pathologist	1	3.4
Oral and Maxillofacial surgery	1	3.4
Radiotherapy specialist	1	3.4
Radiology specialist	1	3.4
Oral and Maxillofacial radiologist	1	3.4
** 10–20 yrs.**	**6**	**20.6**
Oral and Maxillofacial pathologist	1	3.4
Oral and Maxillofacial surgery	1	3.4
Oral and Maxillofacial radiologist	2	6.9
Otorhinolaryngologist	1	3.4
Otorhinolaryngologist - head and neck surgery fellowship	1	3.4
** Less than 5 yrs.**	**1**	**3.4**
Radiotherapy specialist	1	3.4
**Total**	**29**	**100**

### Informational content and practical capabilities extracted from the questionnaire for the design of the registry system

In order to analyze the data and calculate the mean score for each question, the 5-point Likert scale was set as follows: strongly agree = 4, agree = 3, neutral = 2, disagree = 1, and strongly disagree = 0. Based on this scoring, the mean score was calculated for each question. The designed questionnaire had 123 items, of which 114 received an average score higher than 2.5. These 114 items, which are divided into ten main parts, were considered the main information elements of the registry design. Due to the large number of items in the questionnaire, only items with a good mean score (above 2.5) are included in the table. Furthermore, Table 2 shows the scores for selected information elements. The lowest average obtained is related to the item “province and city of parents” and the highest mean score is obtained by a number of items (mean score = 4, n = 20, 17.5%, IQR1: 3.58, median: 3.82, IQR3: 3.91). Fifteen items (13.1%) have an average of 3.86, and 13 items (11.4%) have an average of 3.89. One hundred twelve items (98.2%) have an average score above 3, only two items (1.7%) have an average range of 2.5 to 3, and 94 items (85.9%) have an average above 3.5.

**Table 2:** Mean score and frequency of specialists’ attitude towards the items which are selected for registry design

*Questions of the first part*: *Demographic and clinical information of the patient*	*Strongly agree*	*Agree*	*Neutral*	*Disagree*	*Strongly disagree*	*Mean* *Score* *out* *of 4*
	N (%)	N (%)	N (%)	N (%)	N (%)	
First/last name of patient	18 (51.7)	5 (17.2)	1 (3.4)	3 (10.3)	2 (6.8)	3.17
First/ last name of the patient’s father	13 (44.8)	10 (17.2)	0	6 (20.6)	0	3.03
Sex	21 (72.4)	6 (20.6)	2 (6.8)	0	0	3.58
Birth date	21 (72.4)	6 (20.6)	2 (6.8)	0	0	3.58
Marital status	14 (48.2)	10 (17.2)	0	3 (10.3)	2 (6.8)	3.06
Education level	20 (68.9)	9 (31)	0	0	0	3.68
Job	25 (86.2)	3 (10.3)		1 (3.4)	0	3.79
Height (cm)	17 (58.6)	7 (24.1)	1 (3.4)	0	4 (13.7)	3.13
Weight (kg)	21 (72.4)	6 (20.6)	1 (3.4)	0	0	3.58
BMI	21 (72.4)	6 (20.6)	1 (3.4)	0	0	3.58
Mobile number	23 (79.3)	3 (10.3)	1 (3.4)	0	2 (6.8)	3.58
landline number	18 (62)	5 (17.2)	1 (3.4)	4 (13.7)	0	3.2
Province and city of residence	24 (82.7)	5 (17.2)	0	0	0	3.82
Province and city of birth	23 (79.3)	5 (17.2)	0	1	0	3.72
Province and city of parents	15 (51.7)	5 (17.2)	0	0	8 (27.5)	2.58
Address	18 (62)	9 (31)	0	2 (6.8)	0	3.48
Zip or postal code	16 (55.1)	5 (17.2)	0	0	8 (27.5)	2.72
History of chronic diseases	26 (89.6)	2 (6.8)	0	1 (3.4)	0	3.82
Person’s previous history of cancer	29 (100)	0	0	0	0	4
History of cancer in the family	27 (93.1)	1 (3.4)	0	1 (3.4)	0	3.86
Smoking cigarette history	27 (93.1)	1 (3.4)	0	1 (3.4)	0	3.86
History of hookah use	27 (93.1)	1 (3.4)	0	1 (3.4)	0	3.86
History of alcohol consumption	27 (93.1)	1 (3.4)	0	1 (3.4)	0	3.86
History of drug addiction	28 (96.5)	0	0	1 (3.4)	0	3.89
History of using Naswar	(96.5)28	0	0	1 (3.4)	0	3.89
Organ transplant history	(96.5)28	0	0	1 (3.4)	0	3.89
Oral hygiene status	(96.5)28	0	0	1 (3.4)	0	3.89
***Questions of the second part***: ***Information about drugs***	***Strongly agree***	***Agree***	***Neutral***	***Disagree***	***Strongly disagree***	***Mean*** ***Score*** ***out*** ***of 4***
	*** N (%)***	***N (%)***	***N (%)***	***N (%)***	***N (%)***	
Generic name of used drugs	24 (82.7)	3 (10.3)	0	1 (3.4)	0	3.65
Dose of drug used	19 (65.5)	7 (24.1)	0	3 (10.3)	0	3.44
Frequency of use per day/month	20 (68.9)	6 (20.6)	0	3 (10.3)	0	3.48
How to take the medicine	20 (68.9)	6 (20.6)	0	3 (10.3)	0	3.48
Date of drug prescription	22 (75.8)	6 (20.6)	0	0	1 (3.4)	3.65
End date of drug use	22 (75.8)	6 (20.6)	0	0	1 (3.4)	3.65
***Questions of the third part***: ***initial diagnostic evaluations of the patient***	***Strongly agree***	***Agree***	***Neutral***	***Disagree***	***Strongly disagree***	***Mean*** ***Score*** ***out*** ***of 4***
	*** N (%)***	***N (%)***	***N (%)***	***N (%)***	***N (%)***	
Intraoral radiography	20 (68.9)	5 (17.2)	0	0	3 (10.3)	3.27
Extraoral radiography	20 (68.9)	5 (17.2)	0	0	3 (10.3)	3.27
CT scan of the head and neck	25 (86.2)	3 (10.3)	0	1 (3.4)	0	3.79
CT scan to check systemic metastasis	19 (65.5)	7 (24.1)	0	2 (6.8)	0	3.41
Cone-beam computed tomography systems (CBCT)	16 (55.1)	8 (27.5)	0	0	3 (10.3)	3.03
Magnetic Resonance Imaging (MRI) for diagnosing	18 (62)	7 (24.1)	0	0	2 (6.8)	3.2
Sonography	18 (62)	8 (27.5)	0	0	2 (6.8)	3.31
PET SCAN	20 (68.9)	6 (20.6)	0	2 (6.8)	0	3.44
***Questions of the fourth part***: ***Biopsy***	***Strongly agree***	***Agree***	***Neutral***	***Disagree***	***Strongly disagree***	***Mean*** ***Score*** ***out*** ***of 4***
	*** N (%)***	***N (%)***	***N (%)***	***N (%)***	***N (%)***	
The date of acceptance of the biopsy in the pathology laboratory	24 (82.7)	3 (10.3)	0	2 (6.8)	0	3.68
Date of pathology report	24 (82.7)	3 (10.3)	0	2 (6.8)	0	3.68
Tumor location	27 (93.1)	1 (3.4)	0	1 (3.4)	0	3.86
Tumor involved side	27 (93.1)	1 (3.4)	0	1 (3.4)	0	3.86
Histopathological diagnosis	26 (89.6)	2 (6.8)	0	1 (3.4)	0	3.82
***Questions of the fifth part***: ***Tumor staging at the time of diagnosis***	***Strongly agree***	***Agree***	***Neutral***	***Disagree***	***Strongly disagree***	***Mean*** ***Score*** ***out*** ***of 4***
	*** N (%)***	***N (%)***	***N (%)***	***N (%)***	***N (%)***	
Investigation of tumor spread	26 (89.6)	2 (6.8)	0	1 (3.4)	0	3.82
Evaluation of the status of lymph nodes	29 (100)	0	0	0	0	4
The presence of metastasis at the time of diagnosis	29 (100)	0	0	0	0	4
***Questions of the sixth part***: ***Clinical characteristics of the tumor***	***Strongly agree***	***Agree***	***Neutral***	***Disagree***	***Strongly disagree***	***Mean*** ***Score*** ***out*** ***of 4***
	*** N (%)***	***N (%)***	***N (%)***	***N (%)***	***N (%)***	
Tumor duration in months	26 (89.6)	1 (3.4)	0	2 (6.8)	0	3.82
Clinical appearance of the tumor	26 (89.6)	1 (3.4)	0	1 (3.4)	0	3.72
The exact location of the carcinoma in the mouth	29 (100)	0	0	0	0	4
Tumor Laterality	29 (100)	0	0	0	0	4
Tumor Focality	25 (86.2)	3 (10.3)	0	0	0	3.75
***Questions of the seventh part***: ***Surgery (if done)***	***Strongly agree***	***Agree***	***Neutral***	***Disagree***	***Strongly disagree***	***Mean*** ***Score*** ***out*** ***of 4***
	*** N (%)***	***N (%)***	***N (%)***	***N (%)***	***N (%)***	
Surgery performed	29 (100)	0	0	0	0	4
If surgery is performed: - Date of surgery - Surgical procedure	26 (89.6)	2 (6.8)	0	1 (3.4)	0	3.82
Method of surgery	23 (79.3)	3 (10.3)	0	3 (10.3)	0	3.58
Neck (lymph node) dissection: Therapeutic Prophylactic	29 (100)	0	0	0	0	4
Has jaw reconstruction been done?	23 (79.3)	4 (13.7)	0	2 (6.8)	0	3.65
Any surgery complication	19 (65.5)	4 (13.7)	0	3 (10.3)	0	3.13
***Questions of the eighth part***: ***Histopathological features of the tumor***	***Strongly agree***	***Agree***	***Neutral***	***Disagree***	***Strongly disagree***	***Mean*** ***Score*** ***out*** ***of 4***
	*** N (%)***	***N (%)***	***N (%)***	***N (%)***	***N (%)***	
Date of sample receipt	23 (79.3)	5 (17.2)	0	0	0	3.68
Pathology answer date	24 (82.7)	2 (6.8)	0	1 (3.4)	0	3.55
Tumor Size	29 (100)	0	0	0	0	4
Histological Tumor Type	29 (100)	0	0	0	0	4
Tumor Extension (other structures involved)	27 (93.1)	1 (3.4)	0	1 (3.4)	0	3.86
Specimen Margins	29 (100)	0	0	0	0	4
Depth of Invasion	27 (93.1)	2 (6.8)	0	0	0	3.93
Perineural Invasion	29 (100)	0	0	0	0	4
Worst Pattern of Invasion (WPOI)	27 (93.1)	1 (3.4)	0	1 (3.4)	0	3.86
Regional Lymph Node	29 (100)	0	0	0	0	4
Number of Lymph Nodes with Tumor	29 (100)	0	0	0	0	4
Laterality of Lymph Node(s) with Tumor	29 (100)	0	0	0	0	4
Size of Largest Nodal Metastatic Deposit	26 (89.6)	3 (10.3)	0	0	0	3.89
Extranidal Extension (ENE)	27 (93.1)	2 (6.8)	0	0	0	3.93
Distance of ENE from Lymph Node Capsule	23 (79.3)	5 (17.2)	0	1 (3.4)	0	3.72
Number of Lymph Nodes Examined	29 (100)	0	0	0	0	4
Distant Site (s) Involved, if applicable	25 (86.2)	3 (10.3)	0	0	0	3.75
Primary Tumor (pT)	29 (100)	0	0	0	0	4
Regional Lymph Nodes (pN)	28 (96.5)	1 (3.4)	0	0	0	3.96
***Questions of the nineth part: Radiotherapy (if done)***	***Strongly agree***	***Agree***	***Neutral***	***Disagree***	***Strongly disagree***	***Mean*** ***Score*** ***out*** ***of 4***
	*** N (%)***	***N (%)***	***N (%)***	***N (%)***	***N (%)***	
Has radiotherapy been done?	27 (93.1)	1 (3.4)	0	1 (3.4)	0	3.86
If radiotherapy was performed, what was the reason for its indication?	22 (75.8)	1 (3.4)	0	5 (17.2)	0	3.31
If radiotherapy was indicated but not done, what was the reason?	23 (79.3)	2 (6.8)	0	4 (13.7)	0	3.51
Has chemotherapy been done at the same time? And what was the reason for doing it?	27 (93.1)	1 (3.4)	0	1 (3.4)	0	3.86
If chemotherapy was indicated at the same time as radiotherapy, but it was not done, what was the reason?	27 (93.1)	1 (3.4)	0	1 (3.4)	0	3.86
What is the method of radiotherapy?	27 (93.1)	1 (3.4)	0	1 (3.4)	0	3.86
What is the technique of performing external radiotherapy?	23 (79.3)	2 (6.8)	0	5 (17.2)	0	3.51
Number of scheduled sessions for the patient and the number of sessions received	25 (25.2)	2 (6.8)	0	2 (6.8)	0	3.72
Has there been a break during treatment? If yes, the reason: - Other problems (mention the reason)	27 (93.1)	2 (6.8)	0	0	0	3.93
***Questions of the tenth part***: ***Follow-up information***	***Strongly agree***	***Agree***	***Neutral***	***Disagree***	***Strongly disagree***	***Mean*** ***Score*** ***out*** ***of 4***
	*** N (%)***	***N (%)***	***N (%)***	***N (%)***	***N (%)***	
Date of last contact with the patient	25 (25.2)	4 (13.7)	0	0	0	3.86
Source of patient follow-up	23 (79.3)	5 (17.2)	0	1	0	3.68
Vital status of the patient	27 (93.1)	1 (3.4)	0	1 (3.4)	0	3.86
In case of death, the date of death	27 (93.1)	1 (3.4)	0	1 (3.4)	0	3.86
Was the cause of death cancer?	27 (93.1)	1 (3.4)	0	1 (3.4)	0	3.86
Relapse	29 (100)	0	0	0	0	4
Date of diagnosis of relapse	29 (100)	0	0	0	0	4
If there are subsequent relapses of their history	28 (96.5)	0	0	1 (3.4)	0	3.89
Type of treatment in case of recurrence	28 (96.5)	0	0	1 (3.4)	0	3.89
Date of treatment	28 (96.5)	0	0	1 (3.4)	0	3.89
Detailed information about the presence of metastases in the hand	29 (100)	0	0	0	0	4
In case of metastasis, its location	29 (100)	0	0	0	0	4
If there is metastasis, date of diagnosis	29 (100)	0	0	0	0	4
Type of treatment in case of metastasis	28 (96.5)	0	0	1 (3.4)	0	3.89
Date of treatment	28 (96.5)	0	0	1 (3.4)	0	3.89
***Questions of the eleventh***: ***Evaluation of record quality***	***Strongly agree***	***Agree***	***Neutral***	***Disagree***	***Strongly disagree***	***Mean*** ***Score*** ***out*** ***of 4***
	*** N (%)***	***N (%)***	***N (%)***	***N (%)***	***N (%)***	
The existence of information about the description of the surgery	24 (82.7)	3 (10.3)	0	1 (3.4)	0	3.65
Existence of imaging information (CT, CBCT, MRI, Panoramic view)	26 (89.6)	3 (10.3)	0	0	0	3.89
Presence of biopsy information	26 (89.6)	3 (10.3)	0	0	0	3.89
Biopsy data quality	26 (89.6)	3 (10.3)	0	0	0	3.89
Presence of pathology report	27 (93.1)	2 (6.8)	0	0	0	3.93
Pathology report quality	27 (93.1)	2 (6.8)	0	0	0	3.93
Presence of clinical information	27 (93.1)	2 (6.8)	0	0	0	3.93
Clinical information quality	27 (93.1)	2 (6.8)	0	0	0	3.93
Determination of staging by the doctor in the patient record	26 (89.6)	2 (6.8)	0	1 (3.4)	0	3.72
The quality of the staging insert	26 (89.6)	2 (6.8)	0	1 (3.4)	0	3.72
The existence of patient discharge summary information	26 (89.6)	2 (6.8)	0	1 (3.4)	0	3.72
Quality of discharge summary information	26 (89.6)	2 (6.8)	0	1 (3.4)	0	3.72
Your overall assessment of the quality of the file	23 (79.3)	3	0	1 (3.4)	0	3.51

### Conceptual framework and technical requirements

**Fig. 3:**
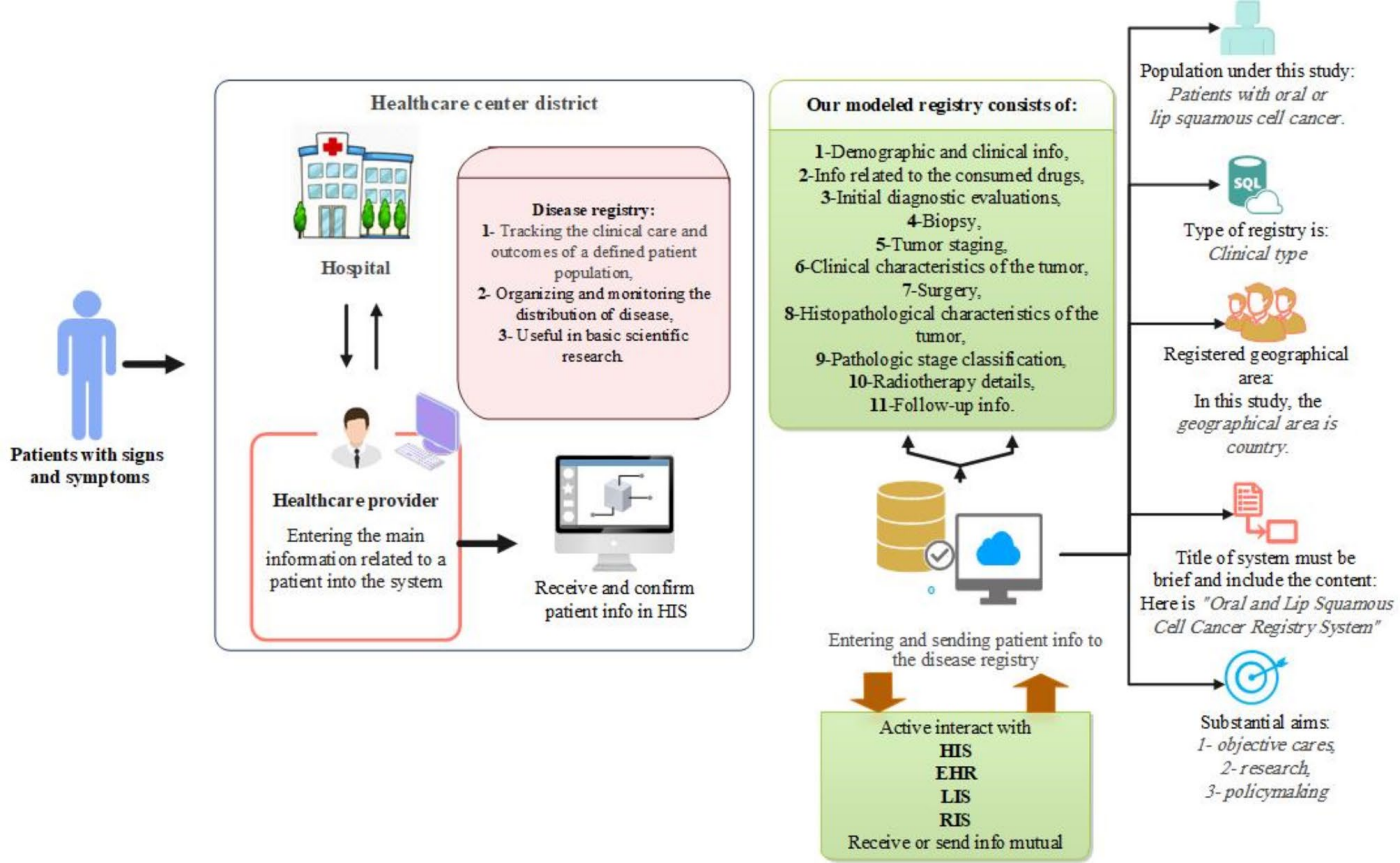
The conceptual framework of the registry

Large databases called clinical registries include data on people who have a certain ailment. As a result, Fig. 3 highlights the distinctive characteristics of the oral and lip squamous cell cancer registry system (specific conceptual framework). The title of the disease registration programme, the population being studied, the geographic extent of the registration, its primary goals, the definition of the condition, the technique of diagnosis, and the kind of registration are all included in this figure.

Some of the technical needs for the oral and lip squamous cell cancer registry system have been determined and are shown in Table 3 based on a comprehensive poll of experts. The external interface specifications needed to create a registry system are listed in this table. At least the number of users, the number of centers, the need for collecting therapeutic-diagnostic data, abstracting and coding, reporting, and active follow-up, compatibility with international standards in data registration (ICD-10), ICD-O, SNOMED CT), and owning interaction with health information systems, ability to create visit schedule and reminder schedule are the main technical requirements of this system.

**Table 3:** Technical specifications for creating a registry for squamous cell carcinoma of the oral and lips

Optional capabilities	Requirement
At least number of users	3
At least number of registration centers	2
Advanced reporting with Excel and SPSS output (data output in various formats)	√
User activity log on each patient record	√
Quality control environment	√
Registration of follow-up referrals	√
Performance reporting in the registration expert environment	√
Performance reporting in the quality control environment	√
High graphics and acceptable usability	√
Several users can use it simultaneously	√
The system must be capable of spatial changes	√
The system must be able to determine the level of users’ access to information	√
Support	√
Ability to create schedule, visit schedule, reminder schedule	√
User training	√
Form design consultation, validations, registration project launch	√
The possibility of entering clinical trial data and comparing the results	√
The system must be compatible with the plans of insurance organizations, hospital information systems.	√
User supplementary package	Three users
The system must be able to exchange information with HIS hospital information systems.	√
The possibility of connecting the patient’s data with the patient’s electronic health record	√
The standards of exchange, terminology, unit, and uniform coding should be used to record and exchange information.	√
The possibility of entering data from different sources	√
The system must be compatible with the Internet	√
Access the system online from any browser	√
Providing reports and graphical analysis for authorized users based on various variables (illustrated reports)	√

## Discussion

The goal of this study was to identify the data needs of the oral and lip squamous cell carcinoma registry system. Eleven major data classes were found after examining the current systems and expert surveys. The described registry system’s eleven primary data classes with capabilities were as follows: 1- demographic and clinical information of the patient, 2-information related to the consumed drugs, 3- initial diagnostic evaluations of the patient, 4- biopsy, 5- tumor staging at the time of diagnosis, 6- clinical characteristics of the tumor, 7-surgery, 8- histopathological characteristics of the tumor, 9- pathologic stage classification, 10- radiotherapy details (if done), 11- follow-up information, and 12- technical capabilities.

The purpose of registering patients in the field of cancer is to control the occurrence and spread of the disease, create a natural course of the disease, monitor and investigate the outcome and survey after treatment, evaluate the clinical effectiveness, measure the quality of care and treatment plan, conduct research according to the cause, and provide It is a source for patients to call back for clinical research [24].

Utilizing registries in the healthcare industry may result in the collection of disease-related data in a standardized and uniform manner [25]. Disease progression and healthcare quality can both be assessed by storing health information in the register [26]. The registers can be used to assess how economically beneficial the treatments are. One of the most important steps in creating and building these systems is figuring out the registry’s minimal data set and information requirements [27–29].

In most cases, the data entered in the registration system is used to plan, implement, and evaluate public health and clinical health activities. Today, many achievements in medical knowledge can be attributed to the analysis and data mining of data collected from patients who have a specific disease [30, 31]. Therefore, the disease’s registry plays a significant role in advancing medical knowledge, new developments in the field of diagnostic and treatment approaches, and they make it easier to provide patients with better health care services and conduct medical research pertaining to a particular disease [32, 33].

In 2022, Akbari et al. conducted a study to establish a minimum data set for managing the data generated during the diagnosis and treatment of oral cancer [34]. They conducted a specialized literature and medical records review and gathered expert opinions, similar to our study. The study proposed a framework to manage data related to the diagnosis and treatment of oral cancer. The framework was divided into six sections: management data with a four-axis, historical data with a four-axis, paraclinical indicators with a two-axis, clinical indicators, data related to the therapeutic measures, and mortality data.

An overview of the National Spinal Cord Injury Registry of Iran (NSCIR-IR) data set development procedure is presented in another paper [35]. Similarly, we were able to create a comprehensive and useful data set thanks to our evidence-based methodology and the review of an interdisciplinary expert panel. To choose the registry data items, some organizations have formed working groups and reviewed the most recent research.

In this study, the registry’s data items were categorized into 12 main classes, which can be further separated into administrative and clinical data categories. The minimal data set for the COVID-19 disease surveillance system was established by a study [36]. In this study, 11 primary classes and 137 fields were identified; the demographic information category had the highest number of data components with 27 fields, followed by the laboratory category with 21 fields. Eleven fields in the current study linked to demographic data, however, the clinical data of the patients received more attention.

Determining a minimal dataset for the COVID-19 registration system was the goal of another study [37]. In line with our study, a qualitative study was conducted to create an MDS for the COVID-19 registration system. The information sources like articles, patient medical records, and opinions that were received from experts (medical specialists) were used for data gathering.

Regarding the importance of registering demographic data in a study conducted in 2014 by Bajraktari and colleagues on 951 patients with rheumatoid arthritis who were registered in the national registry, conducted a survey. The importance of demographic data such as gender, nationality, marital status, level of education, and occupation was proven [38]. It was well determined in our research that there were significant relationships between these items and the prevalence of oral cancer. As a result, for clinical research, where the main purpose of registries is to collect correct and systematic information for this research, the existence of demographic items is effective, and these results are consistent with the results of the present study [39].

In our study, experts have expressed the importance of having information on the clinical involvement of the tumor, biopsy, histopathological features of the tumor, and performing radiotherapy with scores above 3. Also, family history of cancer, history of smoking, hookah, addictive substances such as Naswar, and history of organ transplantation are high-risk factors for oral cancer, and these records are considered in most of the forms designed by European and American countries [40].

In another work [41], a coronary artery disease registration system was designed, and 13 main classes were identified during the process of systematic study and interviews. In this study same as ours, demographic information, history, and risk factors have been identified as the most important elements. The initial review of coronary artery disease (CAD) registry-related sources produced findings that were later reported in two publications. In the following stage, a qualitative study was used to identify the requirements and prerequisites of the software. During this stage, a questionnaire was used to determine the registry dataset. The software’s conceptual model was validated in the end.

In a study, cancer registries have been formed as the cornerstone of a strategy to combat the illness [42]. Similar to our study, owing to this aim, a literature review was conducted; during this review of the texts, the main information elements were identified and finalized by the expert team. A suggested national minimum data set was made in light of the comparative study’s findings. Three subsets of data made up this data set: demographics, tumor and their therapies, and patient death.

In the current study, specialists stated the importance of the existence of treatment information, which included surgical, non-surgical, non-pharmacological, and non-surgical medical information, with scores above 3 as essential. During a study conducted in 2017 on the Life Data from the Lorhen Registry (LORHEN) [43], it was found that in addition to co-morbidities, medical information, including the drugs used, is also important for determining the type of treatment and the primary and secondary treatments of the patient.

Furthermore, in another study conducted in 2006 by Mercer LK et al. on the British Society for Rheumatology Biologics Register (BSRBR), the effect of different treatments and their cost-effectiveness were measured, and these results indicate the importance of registries in this field [44]. In fact, the information related to the provided treatments can provide the basis for studying the effectiveness.

This study had some limitations. Physicians who took part in the focus groups and interviews were also participants in the validation procedure. This may be the probable cause of the high level of agreement in the survey process. The primary structure of the registration system was designed based on the demands of the patients, and this is where our study excels because we first surveyed experts and patients to identify the information elements and functionality of the system.

## Conclusion

In the present study, information requirements for the design and development of an oral cancer registry were identified. The main data classes of this registry are: 1- demographic and clinical information of the patient, 2-information related to the consumed drugs, 3- initial diagnostic evaluations of the patient, 4- biopsy, 5- tumor staging at the time of diagnosis, 6- clinical characteristics of the tumor, 7-surgery, 8- histopathological characteristics of the tumor, 9- pathologic stage classification, 10- radiotherapy details (if done), 11- follow-up information, and 12- disease registry capabilities. Moreover, the conceptual model of the oral registry is provided; the title of the registration programme, the population being studied, the geographic extent of the registration, its primary goals, the definition of the condition, the technique of diagnosis, and the kind of registration are all included in this study. Finally, it can be concluded that the information obtained from MDS provides valuable resources for evaluation, treatment planning, and continuous evaluation of the patient’s progress and performance. The benefits of designing and implementing diseases registries in our country are timely access to medical records, registration of information related to patient care and follow-up of patients, the existence of standard forms and the existence of standard information elements, and the existence of an integrated information system at the country level.

## Data Availability

All data generated or analyzed during this study are included in this published article.
